# Correlations Between LC-MS/MS-Detected Glycomics and NMR-Detected Metabolomics in *Caenorhabditis elegans* Development

**DOI:** 10.3389/fmolb.2019.00049

**Published:** 2019-06-28

**Authors:** M. Osman Sheikh, Fariba Tayyari, Sicong Zhang, Michael T. Judge, D. Brent Weatherly, Francesca V. Ponce, Lance Wells, Arthur S. Edison

**Affiliations:** ^1^Complex Carbohydrate Research Center, University of Georgia, Athens, GA, United States; ^2^Department of Biochemistry and Molecular Biology, University of Georgia, Athens, GA, United States; ^3^Department of Genetics, University of Georgia, Athens, GA, United States; ^4^Institute of Bioinformatics, University of Georgia, Athens, GA, United States

**Keywords:** *C. elegans*, glycomics, mass spectrometry, NMR, metabolomics, development, biosorter

## Abstract

This study examined the relationship between glycans, metabolites, and development in *C. elegans*. Samples of N2 animals were synchronized and grown to five different time points ranging from L1 to a mixed population of adults, gravid adults, and offspring. Each time point was replicated seven times. The samples were each assayed by a large particle flow cytometer (Biosorter) for size distribution data, LC-MS/MS for targeted *N*- and *O*-linked glycans, and NMR for metabolites. The same samples were utilized for all measurements, which allowed for statistical correlations between the data. A new protocol was developed to correlate Biosorter developmental data with LC-MS/MS data to obtain stage-specific information of glycans. From the five time points, four distinct sizes of worms were observed from the Biosorter distributions, ranging from the smallest corresponding to L1 to adult animals. A network model was constructed using the four binned sizes of worms as starting nodes and adding glycans and metabolites that had correlations with *r* ≥ 0.5 to those nodes. The emerging structure of the network showed distinct patterns of *N*- and *O*-linked glycans that were consistent with previous studies. Furthermore, some metabolites that were correlated to these glycans and worm sizes showed interesting interactions. Of note, UDP-GlcNAc had strong positive correlations with many *O*-glycans that were expressed in the largest animals. Similarly, phosphorylcholine correlated with many N-glycans that were expressed in L1 animals.

## Introduction

This paper presents a new approach to evaluate the relationship between *Caenorhabditis elegans* development, glycan abundance, and metabolites. Regardless of the organism, glycomics and metabolomics are generally conducted independently, but metabolism and glycan biosynthesis are intimately related (Freeze et al., 2015). For example, *O*-linked β-*N*-acetylglucosamine (*O*-GlcNAc)—a type of posttranslational glycosylation of nuclear and cytoplasmic proteins—acts as a sensor of nutrition and cellular stress (Zachara and Hart, 2004; Zachara, 2018). The addition of *O*-GlcNAc to proteins is catalyzed by a single enzyme, *O*-GlcNAc transferase (OGT), which relies on the availability of the sugar-nucleotide donor substrate, UDP-GlcNAc via the hexosamine biosynthetic pathway (HBP) (Vaidyanathan and Wells, 2014). Through the HBP, concentrations of UDP-GlcNAc are modulated by the metabolism of glucose, fatty acids, amino acids, and nucleotides. This vital glycosylation precursor is not only utilized by OGT to modify thousands of proteins with *O*-GlcNAc (Zachara and Hart, 2004; Zachara, 2018), but also by many other glycosyltransferases to generate more elaborate types of *N*- and *O*-linked glycans (Brockhausen and Stanley, 2015; Stanley et al., 2015). Better approaches of associating glycomics and metabolomics would be valuable to gain a deeper understanding of their interactions.

*C. elegans* has become an important model organism for chemical signaling (Srinivasan et al., 2008; von Reuss et al., 2012; Ludewig and Schroeder, 2013), metabolism (Srinivasan et al., 2012; von Reuss and Schroeder, 2015; Witting et al., 2018), and glycomics (Paschinger et al., 2008). *C. elegans* has a surprising diversity of many of these groups. Both ascaroside (Srinivasan et al., 2012; von Reuss and Schroeder, 2015) and *O*-GlcNAc (Zachara and Hart, 2004) biosynthesis incorporate many of the same primary metabolic pathways in *C. elegans*. Therefore, *C. elegans* is a good model organism to study the interactions between glycomics and metabolomics. *C. elegans* develops from egg to adult in about 3 days through 4 distinct larval stages (L1-L4), young adult, adult. When resources are limited or the population of worms too high, *C. elegans* enters the dauer stage, which can persist for several months and is specialized for dispersal (Hu, 2007). *C. elegans* development has been studied for decades, including the seminal study that mapped the entire cell lineage of post-embryonic animals and led to the discovery of apoptosis (Sulston and Horvitz, 1977). Gene expression has been linked with development in *C. elegans* through the use of green fluorescent protein (GFP), the first application of this important technique in animals (Chalfie et al., 1994). Metabolites (Srinivasan et al., 2008; Kaplan et al., 2009, 2011) and glycans (Morio et al., 2003; Cipollo et al., 2005; Kanaki et al., 2018) have been implicated in playing major roles in different stages of development. However, because there are no simple tools such as the use of a fluorescent reporter of gene expression, it is still extremely difficult to relate metabolites and glycans to development.

In this study, we used LC-MS/MS to quantify the expression profiles of both *N*- and *O*-linked glycans in *C. elegans* as a function of development. We developed a novel approach to statistically correlate LC-MS/MS glycomics data with Biosorting data, which provides a population distribution of the samples. To our knowledge, until now nobody has statistically associated molecular data such as glycans and metabolites with population distribution data through a large-particle flow cytometer. This allows a direct and unbiased association between glycans and developmental stage. We also collected untargeted NMR data on the same samples used for glycomics and Biosorting. Statistical correlations between LC-MS/MS glycomics and NMR data provided links between specific resonances in the NMR data with specific groups of glycans, which allowed us to begin to interpret the interplay between metabolites and glycans through development in *C. elegans*. Finally, we constructed a correlation network of binned sizes of worms from Biosorter distributions, LC-MS/MS glycomics, and NMR metabolomics data, which exposes some unique interactions between these three distinct types of data.

## Materials and Methods

All data reported in this study have been deposited in the Metabolomics Workbench (doi: 10.21228/M8240W) (Sud et al., 2016).

### Reagents

PNGase A (Protein *N*-Glycosidase A, Calbiochem) was purchased from MilliporeSigma (St. Louis, MO, USA). Sodium hydroxide (50%) was purchased from Fisher Scientific. Sep-Pak C18 disposable extraction columns were obtained from Waters Corporation (Milford, MA, USA). AG-50W-X8 cation exchange resin (H+ form) was purchased from Bio-Rad and trifluoroacetic acid from Pierce. Ultra Pure UDP-GlcNAc was purchased from Promega Corporation (Madison, WI, USA). Trypsin, Chymotrypsin, and all other chemical reagents were purchased from Sigma-Aldrich/MilliporeSigma (St. Louis, MO, USA).

### *C. elegans* Sample Preparation

This study used N2, the laboratory reference strain of *C. elegans*, which was obtained from the Caenorhabditis Genetics Center (CGC). We followed the general protocol published previously for obtaining liquid cultures of synchronized worms (Srinivasan et al., 2008; Kaplan et al., 2009). This defines our biological replicate: A single L1 animal from a synchronized culture was placed onto an agar plate seeded with *E. coli* MG1655. This plate was grown until there were a large number of young gravid adult hermaphrodites (about 48 h at ~24°C). The plate was then washed into a 15 mL tube with M9 buffer, rinsed 3x with M9, and lysed with an alkaline hypochlorite solution until about 50% of the worms were dissolved (no more than 5 min). Then, M9 buffer was added to dilute the lysing solution, and the liquid was removed after gentle centrifugation at 580 g for 2 min to pellet the eggs without breaking them. This step was repeated 3x to completely remove the lysis solution. After the final rinse, eggs were resuspended in sterile water before a sucrose gradient to remove cellular debris and bacteria. An equal volume (5 mL) of 60% sucrose was added to the eggs in water and centrifuged at 350 g for 4 min. The eggs were rinsed to remove residual sucrose and once they hatched, ~200,000 animals were transferred to 20 mL of S-complete with 2 mL of 50% MG1655. This material was grown to the desired developmental stage and prepared as described below.

**Scheme 1:**
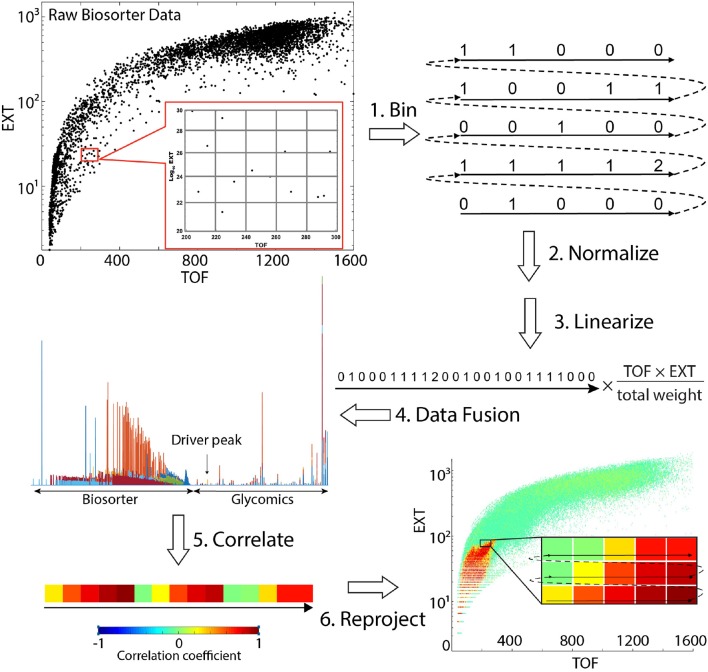
Steps involved in obtaining statistical correlations between mass spectrometry glycomics and Biosorter data. Refer to Correlation Analysis Between Glycomics and Biosorter Data for details.

We started every culture with synchronized L1 arrested animals. To collect animals at different stages, we relied on an approximate number of hours to estimate the stage of the worm population. However, other than L1, we observed that the population had lost some synchrony over time and all worms were not in the same developmental stage at the moment of collection. Isolating large quantities of worms (e.g., 200,000) with uniform stages is not trivial, since there can be residual bacteria and debris after the synchronization step. Therefore, we report results using these time points rather than developmental stages, since they are not all pure stage cultures. The first time, T1, was collected immediately after hatching and were synchronized L1 arrested animals. Therefore, results from T1 can be directly related to L1 stage animals. The relationship between developmental stage and other time points is less clear. Indeed, as time progressed the cultures became more mixed, as shown in Figure S1. The subsequent samples were collected at 22, 36, 49, and 90 h (T2, T3, T4, and T5, respectively) after feeding the cultures. Based on timing and literature values of N2 development, T2 is early larval stages, T3 mid-larval, T4 late-larval to adult, and T5 adults, gravid adults with mixed-stage offspring. These estimates were qualitatively confirmed by visual inspection. To estimate the size of each worm in each sample, we utilized specific ranges of time of flight (TOF) and extinction coefficient (EXT), measured by a large particle flow cytometer called a Biosorter (Union Biometrica). We obtained Biosorter data on each individual sample before homogenization (Figure S1). As described below, we have developed a protocol to recover size-specific information, even from samples that have lost synchrony. This information provides a population distribution and count for each sample, because the location of individual data points in a Biosorter dataset is related to the size and optical density of each worm. This information was then statistically correlated with glycomics and NMR data, as described below (Scheme 1).

### Biosorting

Following worm growth and clean up with sucrose gradient, 2.5% of the cultures were counted and sized using a Union Biometrica Biosorter-Pro large particle flow cytometer using a 250 μm flow cell. Small animals have a shorter time of flight and are optically lighter than larger animals. Therefore, L1 animals are on the lower left of a Biosorter curve and large adults are on the upper right. The Biosorter was calibrated with fluorescent control particles before the runs, as an internal standard. The sheath flow rate was kept constant at 10 mL per minute to decrease variability in length measurements as much as possible. All data were collected using the 488 nm laser with the power set at 50 mW. The signal threshold has set to 500 mV and time of flight minimum was set to 40. Green, yellow, and red photomultiplier tubes were set to 350, 400, and 650 PMT Volts, respectively. All signal gains were set to 1.0. Each Biosorter growth curve is provided in Figure S1.

### Homogenization

The remaining 97.5% of the worm pellets were bead homogenized with 80% methanol/20% water using a FastPrep-24 (MP Biomedicals) for 5 cycles of 60 s. The tubes were then centrifuged at high speed for 20 min to separate the supernatant from the beads. This process was repeated twice, and the supernatants were combined. The supernatant was then placed in a Labconco SpeedVac until no liquid was observed in the sample. The dried supernatant was then stored at −80°C until NMR analysis. The pellets with beads were frozen at −80°C until glycomics analysis.

### NMR Data Collection and Analysis

NMR spectroscopy is a relatively low sensitivity measurement that requires samples with concentrations <about 10 μM for detection. Early larval stage animals are considerably smaller than adults and contribute much less mass per worm. Therefore, the supernatants of the 7 T1 time points were combined into one sample for the NMR analysis. This resulted in 29 dried extracted-worm pellets, which were dissolved in 600 μL NMR buffer (0.1 M sodium phosphate buffer in D_2_O with a final concentration of 0.33 mM of DSS) and mixed well using a vortex mixer. Five hundred and ninety microliter of each sample were added to 5 mm NMR tubes.

NMR data were collected at 600 MHz on a Bruker AVIII-HD console in a magnet equipped with a 5 mm CryoProbe and a SampleJet autosampler, which cooled samples to 6°C while waiting in the queue. All NMR acquisitions were performed at 27°C (300 K). One dimensional (1D) nuclear Overhauser enhancement spectroscopy with presaturation (NOESY-PR) was obtained on each sample, and two-dimensional ^13^C-^1^H heteronuclear single quantum coherence (HSQC) and ^13^C-^1^H heteronuclear single quantum coherence-total correlation spectroscopy (HSQC-TOCSY) were obtained from one representative sample from each time point for compound identification.

NMR data were processed with NMRPipe using standard parameters (Delaglio et al., 1995). For post processing, we used a MATLAB metabolomics toolbox developed in the Edison laboratory (Robinette et al., 2011). Spectra were referenced to the DSS resonance at 0.0 ppm, and regions corresponding to water and methanol were removed. Aligning NMR peaks can be a challenge. Chemical shifts are sensitive to several factors including pH, sample matrix, and/or ion contents. Different alignment algorithms were compared to find a proper alignment but none of them was perfect for all the regions of the spectra. Therefore, we combined results of three different peak alignment algorithms. We normalized each alignment using probabilistic quotient normalization (PQN) (Dieterle et al., 2006). Constrained correlation optimized warping (CCOW) (Nielsen et al., 1998) using spearman cluster method was best for the first region from 0 to 3.3566 ppm, CCOW with correlation cluster method was best for the region between 3.3566 and 4.7651ppm, and Fast Fourier transform (PAFFT) (Wong et al., 2005) with correlation cluster method best for the remainder. The best regions from each method were then combined. Statistical total correlation spectroscopy (STOCSY) was used both to find the correlation between the intensities of the different peaks across the whole sample to aid in NMR identification (Cloarec et al., 2005) and also to correlate specific NMR peaks with glycans (Crockford et al., 2006).

For metabolite identification we used a combination of COLMARm (Bingol et al., 2016) and spiking. For spiking experiments, we first obtained a spectrum of the sample before spiking, along with a separate spectrum of the authentic metabolite standard. Then a small quantity of the authentic metabolite was added into the sample and NMR data recorded. The compound ID was verified if the authentic and putative NMR signals add together with no additional resonances in the spiked spectrum. We used a confidence scale for metabolite ID that ranges from 1 (lowest) to 5 (highest): (5) verified by spiking; (4) Matches in COLMARm using both HSQC and HSQC-TOCSY; (3) COLMARm matches using HSQC but not HSQC-TOCSY; (2) matches from 1D NMR to literature and/or database libraries such as BMRB (Ulrich et al., 2008) or HMDB (Wishart et al., 2007); (1) for putatively characterized compounds or compound classes.

### Interactive Binning of NMR Data

For the creation of the correlation network between NMR resonances, glycans, and worm sizes (**Figure 5B**, results), the results were too complicated using full-resolution NMR data. Since we were interested in correlations between specific NMR features and the network, automatic binning using a uniform bin size across the spectrum would not yield the desired results. Therefore, we developed a new workflow in MATLAB that allows for interactive binning of features of interest from an NMR dataset. This results in features of arbitrary width and with bins with user-specified boundaries. An example region of this interactive binning is shown in Figure S3.

Starting from a working directory with the spectral matrix and ppm vector loaded in MATLAB, the first block of code initializes parameters and creates directories to store results. The second block is then run. If there is no figure already provided as input in “figureFileNames,” a new overlay plot is generated from the matrix and ppm vectors provided. The user zooms to the next peak of interest, then clicks a button in the window to draw rectangular boundaries for a feature, which is then highlighted. Boundaries from different features can overlap partially or completely, and only the left and right boundaries are considered. A prompt allows the user the option of choosing another feature or ending the workflow. When all features have been selected, the user responds “N,” and the program saves the result as a.fig file as well as in an updated variable called “ROIs.” In addition, a running list of.fig filenames is kept by the script. For speed purposes (as well as security of saving progress), we save after every ~30-50 features and recommend going across the spectrum in a linear fashion. The second block is run repeatedly until all features have been recorded (for ~700 features, typically 3–5 h). The third block of code compiles the features from all the figures and produces a.fig file containing these. Regions are integrated by summing all intensities between feature boundaries and are reported in the “features” structure object. These are overlaid on the spectra and used in downstream statistical analyses. Each feature is also assigned a unique name, which is the closest unused ppm value to the maximum within the feature boundaries. This workflow is available on the Edison Lab GitHub site (https://github.com/artedison/Edison_Lab_Shared_Metabolomics_UGA).

### Glycomics Sample Preparation

Using the frozen extracted pellets from homogenization described above, we further delipidated by resuspending the pellets in chloroform/methanol/water (4:8:3, v/v/v) as described previously (Aoki et al., 2007). Insoluble proteins were pelleted by centrifugation, and protein pellets were washed twice with ice-cold acetone. Finally, protein power was dried under a nitrogen evaporator.

Preparation of glycopeptides and release of *N*-linked glycans was performed as described previously (Aoki et al., 2007). Briefly, approximately 5 mg of protein powder from each sample was resuspended in 500 μL of 40 mM NH_4_HCO_3_, 1 M urea, 20 μg/mL trypsin, and 20 μg/mL chymotrypsin and incubated overnight (16–18 h) at 37°C. The glycopeptide mixture was boiled for 5 min and adjusted to 5% AcOH (acetic acid) prior to a Sep-Pak C18 cartridge column clean up. Glycopeptides were eluted stepwise in 20% isopropanol in 5% AcOH, 40% isopropanol in 5% AcOH, and 100% isopropanol. The eluates were pooled and evaporated to dryness. Dried glycopeptides were resuspended in 50 mM citrate phosphate buffer (pH 5.0) for digestion with peptide:*N*-glycosidase A (PNGase A) and incubated for 18 h at 37°C. We chose to utilize PNGase A for the release of *N*-linked glycans since its substrate-specificity is less stringent than the commonly used PNGase F. Specifically, PNGase A is capable of releasing *N*-glycan species containing α1-3-linked fucose on the chitobiose core, known to be synthesized by *C. elegans* (Paschinger et al., 2008; Schachter, 2009). PNGase A-released oligosaccharides were separated from residual peptides by another round of Sep-Pak C18 cartridge clean-up, and the glycan flow-through was collected. Released *N*-glycans were dried down using a SpeedVac.

Since there is currently no available enzyme for the comprehensive release of *O*-linked glycans, we employed a commonly used chemical release strategy via reductive β-elimination using NaOH and NaBH_4_ (von Reuss and Schroeder, 2015). Approximately 5 mg of protein powder from each sample was processed for reductive β-elimination to release *O*-linked glycan alditols as described previously (Aoki et al., 2008). Briefly, protein powder was resuspended in 100 mM NaOH containing 1 M NaBH_4_ and incubated for 18 h at 45°C in a glass tube sealed with a teflon-lined screw top. Following incubation, the protein concentration of the reaction mixture was determined via absorbance at 280 nm using a NanoDrop ND-1000 spectrophotometer (NanoDrop Technologies, Wilmington, DE). For normalization of all samples, 2 mg of the reaction mix was neutralized with 10% acetic acid on ice and desalted using a AG-50W-X8 (H^+^ form) column (1 mL bed volume) prior to borate removal and Sep-pack C18 cartridge clean-up. Released *O*-glycans were dried down using a SpeedVac.

Both *N*- and *O*-glycans were permethylated to introduce hydrophobicity, fragmentation, and facilitate in-line separation by reverse-phase (C18) chromatography prior to detection by mass spectrometry (MS) (Brockhausen and Stanley, 2015; Stanley et al., 2015). Furthermore, permethylation of glycans greatly improves MS ionization efficiency resulting in improved quantification. All released *N*- and *O*-linked glycans were permethylated prior to MS analysis according to the method by Anumula and Taylor (Anumula and Taylor, 1992).

### NanoLC-MS/MS of Permethylated Glycans

Dried down neutral/non-sulfated permethylated glycans were resuspended in 100 μL of 100% methanol. Samples were prepared by combining 4 μL of resuspended glycans with 4 μL of an internal standard [^13^C-Permethylated isomaltopentaose (DP5)] at a final concentration of 0.2 pmol/μL and 32 μL of LC-MS Buffer A (1 mM LiOAc and 0.02% acetic acid). For each LC-MS/MS analysis, 5 μL of each prepared sample was injected for liquid chromatography separation using an Ultimate 3000 RSLC (ThermoFisher Scientific/Dionex) equipped with a PepMap Acclaim analytical C18 column (75 μm ×15 cm, 2 μm pore size) coupled to a ThermoScientific Velos Pro Dual-Pressure Linear Ion Trap mass spectrometer. The HPLC column oven temperature was set to 60°C to achieve optimal separation of permethylated glycans. After equilibrating the column in 99% LC-MS Buffer A for 5 min, separation was achieved using a linear gradient from 30% to 70% LC-MS Buffer B over 150 min at a flow rate of 300 nL/min. The analytical column was regenerated after each run by washing in 99% LC-MS Buffer B for 10 min and then returning to 99% LC-MS Buffer A for re-equilibration. Spray into the mass spectrometer using nanospray ionization in positive ion mode was via a stainless-steel emitter with spray voltage set to 1.8 kV and capillary temperature set at 210°C. The MS method consisted of first collecting a full ITMS (MS1) survey scan, followed by MS2 fragmentation of the Top 10 most intense peaks using CID at 50% collision energy using an isolation window of 2 m/z. Dynamic exclusion parameters were set to exclude ions for fragmentation for 15 s if they were detected and fragmented 5 times in 15 s.

### Analysis of NanoLC-MS/MS Data

Lists of candidate *N*- and *O*-glycan compositions known to be expressed in *C. elegans* were generated based previous reports (Guerardel et al., 2001; Cipollo et al., 2002, 2005; Natsuka et al., 2002; Paschinger et al., 2008; Schachter, 2009; Geyer et al., 2012; Parsons et al., 2014). Glycan compositions known to be natively methylated in *C. elegans* were intentionally omitted in our targeted database as that information would be lost through permethylation. Phosphorylcholine-modified *N*-glycans were not considered in our study for simplicity. Glycan isomer abundances of a specific composition were summed as a single species. For each MS run, only scans after the 20 min mark (after column equilibration and sample loading) were considered. For each candidate glycan, MS/MS scans were identified where the precursor *m/z* was within 3 Da of the candidate *m/z*, considering charge states (*z*) of +1, +2, and +3. The background intensity of the precursors was calculated by first determining the max precursor intensity (maxPreInt) or all precursors matching a particular candidate glycan (numPrecursors), binning the precursor intensities to create an intensity distribution where the number of bins was equal to maxPreInt / numPrecursors, and then determining the value at the 15th percentile of the distribution to represent the background intensity (bgInt). All MS/MS scans with precursor intensity <1.5 × bgInt were discarded. The total ion count (TIC) for each glycan was calculated by summing the intensities from each peak in the assigned MS/MS scans in each MS run (glycanTIC).

A two-fold normalization method was utilized across the 35 runs for each sample type (*N*- and *O*-glycans) as follows. For each MS run, the sum of the TIC for all glycans was calculated (repSumTIC). The max replicate TIC sum (maxRepSumTIC) was determined for each time point. A normalization factor (repNormFactor) was determined for each replicate as repSumTIC / maxRepSumTIC for each time point. For the internal standard glycan (^13^C-permethylated isomaltopentaose, DP5), the intensity for each replicate was set to the maximum DP5 glycanTIC intensity over all replicates for each time point, under the assumption that an equal amount of that glycan is present in each replicate. For the first normalization, the glycanTIC was multiplied by the repNormFactor (glycanTIC × repNormFactor) for all experimental glycan assignments for each replicate to calculate the normalized glycan TIC (glycanNormTIC). For the second normalization, the final glycan intensity value (glycanNormTICFinal) was calculated by dividing the normalized glycan TIC intensity by the standard normalized intensity (glycanNormTIC / stdNormTIC). Symbol and Text nomenclature for representation of glycan structures is displayed according to the Symbol Nomenclature for Glycans (SNFG) (Varki et al., 2015).

### Glycan Clustering

Hierarchical Clustering Analysis (HCA) of glycans was performed using the MATLAB built-in function “clustergram.” Mean quantities of glycans across replicates of the same time point were calculated and imported for clustering analysis. Data were clustered in both dimensions using Euclidean distance. Linkages were calculated according to the average Euclidean distance of the new cluster. Data were standardized only on the row dimension.

### Correlation Analysis Between Glycomics and Biosorter Data

Scheme 1 provides a visual summary of the steps involved in correlating analytical LC-MS/MS (or NMR) data with worm size in *C. elegans*. The overall strategy is to calculate the statistical correlation of a specific analytical feature (e.g., glycan) to the Biosorter data (or *vice versa*) across all samples. The data required include (1) raw Biosorter data, (2) normalized analytical data, and (3) the sample run order that relates the two datasets. We created a new MATLAB workflow that is freely available through the Edison lab GitHub site for this analysis. The steps below correspond to the steps in Scheme 1.

#### Bin Biosorter Data

Raw data from the Biosorter were read and processed in MATLAB by an in-house script. The Biosorter instrument measures time of flight (TOF), which can be interpreted as the worm's length, and extinction coefficient (EXT), or optical density. Each individual event (e.g., worm) in a Biosorter plot is a separate point in the 2D TOF vs. Log_10_ EXT plots. Thus, in addition to each worm's length and optical density, these plots provide an accurate count of the number of worms in each sample. The individual Biosorter plots for each sample in this study are shown in Figure S1. The first step was to bin the Biosorter plots in both TOF and EXT dimensions simultaneously for each sample so that each bin counted the number of worms in both dimensions. The bin size can be adjusted, but we have found consistently good results with 1 unit as the binning size in both dimensions, the settings that are hardcoded into the script. For a given study, the bin sizes are constant. This step yields an *m* by *n* matrix for each sample containing the number of worms counted, where *m* is the number of bins along the TOF axis and *n* the number of bins along the EXT axis. We excluded Biosorter data that were beyond 1,600 units in both dimensions, because larger numbers are artifacts caused by clumps of material or multiple worms.

#### Normalize by Worm Mass

Mass spectrometry or NMR spectroscopy are both dependent on sample mass, and the mass of an individual worm varies considerably from L1 to adult. Therefore, we normalized the counts in each bin by multiplying the estimated mass of the worms in each bin. There are several possible ways of doing this, including making detailed experimental mass measurements of known numbers of worms at each developmental stage. We chose to use a simpler approach: neglecting bent worms traveling through the flow cell, the TOF measurements are proportional to the length of each worm. Without a specific fluorescent marker, the EXT measurement is proportional to the thickness of the worm (larger worms are less transparent than smaller worms). We multiplied the means of TOF and EXT for each bin and assume that this is proportional to the mass of each worm. Then, to correct for differences in total numbers of worms between replicates, we also normalized each sample by total mass. To do this, we calculated the total estimated worm weight in each sample by summing up the estimated worm weights across all bins. The normalized counts were divided by the total weight of the worms in that sample.

#### Linearize the Biosorter 2D Matrix

To statistically correlate the Biosorter with glycomics data, we first converted the binned 2D (TOF vs. EXT) matrix to a 1D vector. This step was previously developed by the Edison lab for general 2D NMR multivariate analysis (Robinette et al., 2011). This step simply involves extracting rows from the binned and normalized 2D matrix and combining them as indicated in Scheme 1.

#### Data Fusion

The linearized 1D vectors from step 3 were joined with the corresponding glycomics LC-MS vectors to make a single vector with Biosorter and LC-MS data from the same sample. For this step, any type of quantitative analytical data can substitute for the glycomics data used here. The overall concept for this step is an extension of statistical heterospectroscopy (SHY) (Crockford et al., 2006).

#### Correlate the Data

STOCSY (Cloarec et al., 2005) is a statistical method that essentially correlates all points in a sample set of vectors with a specific point along the vectors, which is termed the “driver peak.” We modified the standard STOCSY script in our MATLAB metabolomics toolbox to perform the correlation analysis. The linearized vectors generated in step 4 were imported, along with a modified X-axis vector that assigns an arbitrary scale to specify the driver peak. We also specify a threshold for the resulting correlation coefficients. We then calculated Pearson correlation coefficients between the driver peak and the rest of the data. The inverse correlation can be also calculated by specifying a 2D region of interest in the Biosorter plot as the driver peak to find all glycans that correlate to a specific developmental stage.

#### Reproject the Statistical Correlations Onto a 2D Biosorter Plot

The output of step 5 is a single vector with correlation values to the driver peak. To easily visualize the correlations, the vector was first separated into the Biosorter and glycomics components and the linearization in step 3 reversed. This results in a plot that superficially resembles the original Biosorter data (Figure S1) but now represents the Pearson correlation values to the driver peak from a specific glycan. Thus, the final output from this procedure is a 2D Biosorter map that is the statistical correlation between a specific glycan driver peak and the worm population. In the example figure shown in Scheme 1, the bright red region corresponds to small animals and indicates that the selected driver peak from glycan analysis is highly correlated to that size of worms.

### Building a Correlation Network

We used Cytoscape (v. 3.7.1) (Shannon et al., 2003) to generate a correlation network between NMR features, glycans, and approximate sizes of worms. We found that if we included all the full-resolution NMR features, the network was uninterpretable. Therefore, we used the interactive binning algorithm for NMR data described above. Even with the binned data, the highly correlated NMR data dominated the network, so we first filtered the NMR bins for those that had robust correlations between specific glycans. This was done using SHY (Crockford et al., 2006), similar to the Biosorter correlations with glycans described above. Select NMR statistical correlations to each glycan are provided in Figure S2. We picked the highly correlated NMR features to include as binned data in the Cytoscape network.

As described above, without detailed image analysis, it is difficult to assign a specific developmental stage of a worm to a region of a Biosorter plot. Therefore, for this study we hand-selected different non-overlapping regions from a Biosorter distribution that correspond to different sizes (TOF) and optical densities (Log_10_ EXT). We overlaid the Biosorter time points of Figure S1 as a guide to bin four distinct regions that corresponded to different sizes of worms (results, **Figure 5A**). We summed the total normalized worm counts within each of these regions to include in the Cytoscape network.

Pearson correlation coefficients were calculated in MATLAB between each glycan, the binned NMR features, and the sum of worm counts in each binned Biosorter region. Because we were evaluating correlations between different classes of molecular species along pathways and associated with size, we explored a range of values and empirically found that a threshold of |r| ≥ 0.5 provided a network with multiple nodes and edges that was simple enough to interpret. The *p*-values for this network ranged from 0.005745 to 3.3^*^10^∧^−21, indicating that all were statistically significant correlations. The correlation coefficients table with absolute values greater than or equal to 0.5 was exported from MATLAB to Cytoscape (3.7.1). We started by coloring all edges red for positive and blue for negative values of Pearson correlations and setting the linewidths to 0.5. We then highlighted interactions to each Biosorter region by manually selecting one of the four Biosorter worm sizes, automatically selecting its nearest neighbors, and setting the linewidth to 10.0 of the edges between this subnetwork. After doing this for all four Biosorter regions, we could easily visualize direct correlations from all nodes to each Biosorter region. We then manually organized the network by clustering directly correlated nodes. We also highlighted specific interactions of two NMR metabolites, phosphorylcholine and UDP-GlcNAc, as described in the caption for **Figure 5B**.

## Results

**Figure 1:**
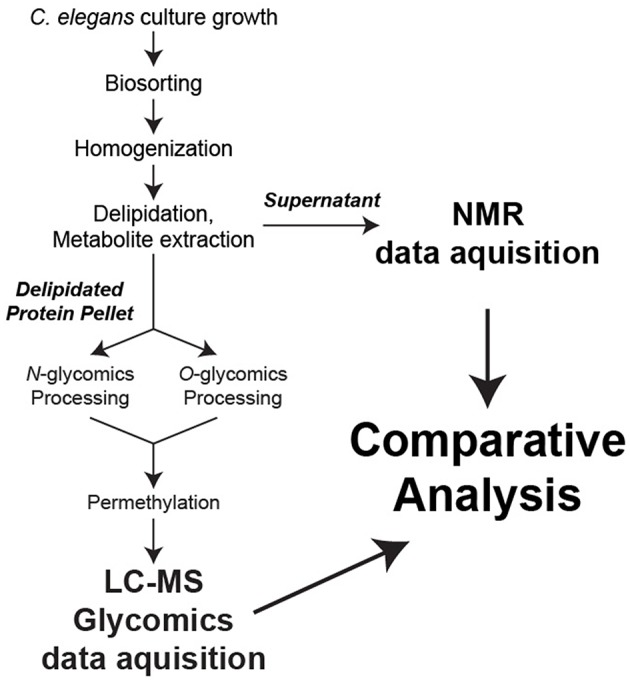
Sample preparation workflow. For each biological replicate, metabolites were extracted for NMR analysis while *N*- and *O*-linked glycans were released from total protein and permethylated for LC-MS analysis.

In an effort to establish novel connections between the glycome and metabolome of *C. elegans* at defined worm sizes, we developed a strategy to utilize a single biological replicate for all stages of sample preparation, data collection, and analysis (Figure 1).

### *N*-Glycan Abundances Cycle During *C. elegans* Development

Using a targeted approach, we generated a list of known *N*-glycans reported in the literature for the wild-type N2 strain (Cipollo et al., 2002, 2005; Haslam et al., 2002; Natsuka et al., 2002; Haslam and Dell, 2003; Paschinger et al., 2008; Schachter, 2009). Glycan compositions known to be natively methylated in *C. elegans* were omitted in our targeted list as native methylation would be masked when glycans are derivatized through permethylation. Additionally, while *C. elegans* is known to modify the core and termini of hybrid- and complex-type *N*-glycans with phosphorylcholine (PC) (Cipollo et al., 2002, 2005), these low abundance structures were omitted from the target list for simplicity of analysis. To the best of our knowledge, previously reported *N*-glycomic data [reviewed in (Paschinger et al., 2008)] derives from mixed stage worms except for one study by Cipollo et al. (2005). Thus, our study aims to identify global variations in the glycome that may exist between different sizes of worms.

**Figure 2:**
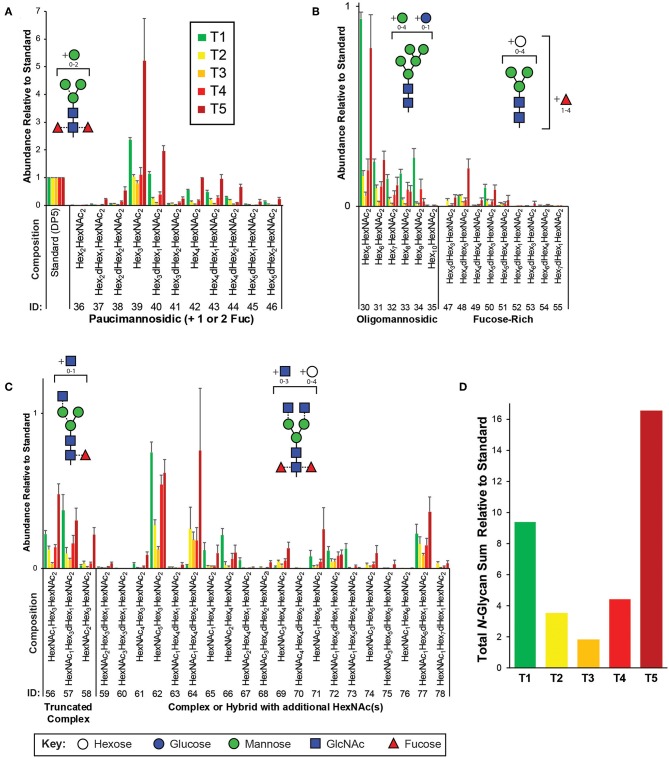
Targeted *N*-glycomics of *C. elegans* at various developmental time points. Presented are the relative abundances of indicated *N*-glycan compositions. Specific *N*-glycan subgroups are indicated in the following panels: **(A)** Paucimannosidic **(B)** Oligomannosidic/Fucose-rich **(C)** Truncated complex/Complex/Hybrid. Representative cartoon examples of each subgroup structure are shown as insets and displayed in accordance with the SNFG guidelines (Varki et al., 2015). Glycan IDs are presented in Table 1. *N*-glycans are presented with the chitobiose core disaccharides (GlcNAc_2_ or displayed as HexNAc_2_) written toward the right. The sum for the relative *N*-glycan abundances at each time point is presented in **(D)**. Each bar represents the sample mean relative to the internal standard (^13^C-permthylated isomaltopentaose, DP5) where *n* = 7 and the error bars represent Standard Error of the Mean (SEM).

The criteria for identification and quantification of candidate glycans by LC-MS/MS are presented in *Materials and Methods*, and glycan isomers of a specific composition were treated as a single species. For each biological replicate, glycoprotein starting material was normalized by mass prior to glycan release and derivatization. Glycan compositions identified in this study that are most consistent with specific glycan subgroups are summarized in Table 1 and Figure 2. Identification numbers were assigned arbitrarily for ease of presentation. Since epimers of carbohydrate residues have indistinguishable masses, we have reported glycan compositions detected by mass spectrometry using the generalized convention as follows, with the named epimers (and abbreviations in parentheses) specific to what has been documented for *C. elegans* previously: **Hex** [hexose, either glucose (Glc), galactose (Gal), or mannose (Man)], **HexNAc** [*N*-acetylhexosamine, either *N*-acetylglucosamine (GlcNAc) or *N*-acetylgalactosamine (GalNAc)], **dHex** [deoxyhexose, namely fucose (Fuc)], and **HexA** [hexuronic acid, namely glucuronic acid (GlcA)].

**Table 1:** Glycan compositions identified in this study.

**Identifier**	**Glycan composition**	**Glycan subgroup**	**Calculated mass (Permethylated)**	**Observed *m/z* (Permethylated, lithiated)**
**O-GLYCANS (REDUCED REDUCING END)**				
1	Hex_1_HexNAc_1_	Neutral	511.3	518.4
2	Hex_2_HexNAc_1_		715.4	722.4
3	Hex_3_HexNAc_1_		919.5	926.5
4	Hex_4_HexNAc_1_		1123.6	568.7
5	HexA_1_Hex_1_HexNAc_1_	Charged	729.4	736.4
6	HexA_1_Hex_2_HexNAc_1_		933.5	940.6
7	HexA_1_Hex_3_HexNAc_1_		1137.6	575.6
8	HexA_1_Hex_4_HexNAc_1_		1341.7	677.6
9	dHex_1_Hex_1_HexNAc_1_	Neutral + Fucose(s)	685.4	692.5
10	dHex_1_Hex_2_HexNAc_1_		889.5	896.5
11	dHex_1_Hex_4_HexNAc_1_		1297.7	655.6
12	dHex_2_Hex_4_HexNAc_1_		1471.8	742.7
13	dHex_2_Hex_5_HexNAc_1_		1675.9	565.4
14	dHex_2_Hex_2_HexNAc_2_		1308.7	661.5
15	dHex_1_Hex_3_HexNAc_1_		1093.6	553.7
16	HexA_1_dHex_1_Hex_1_HexNAc_1_	Charged + Fucose(s)	903.5	910.5
17	HexA_1_dHex_1_Hex_2_HexNAc_1_		1107.6	560.7
18	HexA_1_dHex_1_Hex_3_HexNAc_1_		1311.7	662.7
19	HexA_1_dHex_1_Hex_2_HexNAc_2_		1352.7	683.5
20	HexA_1_dHex_1_Hex_3_HexNAc_2_		1556.8	785.6
21	HexA_1_dHex_2_Hex_1_HexNAc_2_		1322.7	668.5
22	HexA_1_dHex_2_Hex_2_HexNAc_2_		1526.8	770.6
23	HexA_1_dHex_3_Hex_2_HexNAc_2_		1700.9	574.1
24	HexA_1_dHex_3_Hex_3_HexNAc_2_		1905.0	959.8
25	HexA_1_dHex_4_Hex_3_HexNAc_2_		2079.1	1046.8
26	HexA_1_dHex_4_Hex_4_HexNAc_2_		2283.2	767.9
27	HexA_1_dHex_4_Hex_5_HexNAc_2_		2487.3	836.4
28	HexA_1_dHex_5_Hex_4_HexNAc_2_		2457.3	826.3
29	HexA_1_dHex_5_Hex_5_HexNAc_2_		2661.4	894.2
**N-GLYCANS (FREE REDUCING END)**				
30	Hex_5_HexNAc_2_	Oligomannosidic	1556.8	785.6
31	Hex_6_HexNAc_2_		1760.9	887.3
32	Hex_7_HexNAc_2_		1965.0	989.7
33	Hex_8_HexNAc_2_		2169.1	1091.8
34	Hex_9_HexNAc_2_		2373.2	798.4
35	Hex_10_HexNAc_2_		2577.3	866.2
36	Hex_2_HexNAc_2_	Paucimannosidic (+ 1 or 2 Fucoses)	944.5	951.6
37	Hex_2_dHex_1_HexNAc_2_		1118.6	566.5
38	Hex_2_dHex_2_HexNAc_2_		1292.7	653.5
39	Hex_3_HexNAc_2_		1148.6	581.4
40	Hex_3_dHex_1_HexNAc_2_		1322.7	668.5
41	Hex_3_dHex_2_HexNAc_2_		1496.8	755.6
42	Hex_4_HexNAc_2_		1352.7	683.6
43	Hex_4_dHex_1_HexNAc_2_		1526.8	770.6
44	Hex_4_dHex_2_HexNAc_2_		1700.9	857.7
45	Hex_5_dHex_1_HexNAc_2_		1730.9	872.7
46	Hex_5_dHex_2_HexNAc_2_		1905.0	959.8
47	Hex_3_dHex_3_HexNAc_2_	Fucose-Rich	1670.9	842.7
48	Hex_4_dHex_3_HexNAc_2_		1875.0	944.7
49	Hex_4_dHex_4_HexNAc_2_		2049.1	1031.5
50	Hex_5_dHex_3_HexNAc_2_		2079.1	700.0
51	Hex_5_dHex_4_HexNAc_2_		2253.2	758.1
52	Hex_6_dHex_1_HexNAc_2_		1935.0	974.7
53	Hex_6_dHex_3_HexNAc_2_		2283.2	768.3
54	Hex_6_dHex_4_HexNAc_2_		2457.2	826.4
55	Hex_7_dHex_1_HexNAc_2_		2139.1	720.3
56	HexNAc_1_Hex_3_HexNAc_2_	Truncated Complex	1393.7	703.7
57	HexNAc_1_Hex_3_dHex_1_HexNAc_2_		1567.8	529.3
58	HexNAc_2_Hex_3_HexNAc_2_		1638.8	826.7
59	HexNAc_2_Hex_3_dHex_1_HexNAc_2_	Complex or Hybrid with additional HexNAc(s)	1812.9	611.4
60	HexNAc_3_Hex_3_dHex_1_HexNAc_2_		2058.1	693.3
61	HexNAc_4_Hex_3_HexNAc_2_		2129.1	716.5
62	HexNAc_5_Hex_3_HexNAc_2_		2374.2	798.4
63	HexNAc_1_Hex_4_dHex_1_HexNAc_2_		1771.9	597.4
64	HexNAc_1_Hex_4_dHex_2_HexNAc_2_		1946.0	655.4
65	HexNAc_1_Hex_4_HexNAc_2_		1597.8	539.4
66	HexNAc_2_Hex_4_HexNAc_2_		1842.9	621.3
67	HexNAc_2_Hex_4_dHex_1_HexNAc_2_		2017.0	679.5
68	HexNAc_3_Hex_4_dHex_2_HexNAc_2_		2436.3	818.8
69	HexNAc_3_Hex_4_HexNAc_2_		2088.1	703.2
70	HexNAc_4_Hex_4_dHex_2_HexNAc_2_		2681.4	900.5
71	HexNAc_1_Hex_5_HexNAc_2_		1801.9	607.4
72	HexNAc_1_Hex_5_dHex_1_HexNAc_2_		1976.0	665.5
73	HexNAc_1_Hex_5_dHex_2_HexNAc_2_		2150.1	723.5
74	HexNAc_3_Hex_5_HexNAc_2_		2292.2	770.8
75	HexNAc_3_Hex_5_dHex_2_HexNAc_2_		2640.4	886.9
76	HexNAc_1_Hex_6_HexNAc_2_		2006.0	675.5
77	HexNAc_1_Hex_6_dHex_1_HexNAc_2_		2180.1	733.4
78	HexNAc_1_Hex_7_dHex_1_HexNAc_2_		2384.2	801.6

*Hex, Hexose; HexNAc, N-acetylhexosamine; dHex, Deoxyhexose; HexA, Hexuronic acid*.

*N-glycans are presented with the chitobiose core disaccharides (GlcNAc_2_ or displayed in the table as HexNAc_2_) written toward the right*.

The most abundant structures identified belong to the paucimannosidic (dHex_0−2_Hex_2−4_HexNAc_2_) and oligomannosidic (also known as “high-mannose” containing Hex_5−10_HexNAc_2_) subgroups, consistent with previous reports (Figures 2A,B) (Cipollo et al., 2002; Haslam et al., 2002; Natsuka et al., 2002; Paschinger et al., 2008; Geyer et al., 2012). Of the paucimannose structures, which are atypical in vertebrates, the trimannosyl core structure (#39) and its monofucosylated derivative (#40) are of the greatest abundance. High levels of Hex_5_HexNAc_2_ (#30) were detected, with lesser amounts of the larger Hex_6−10_HexNAc_2_ (#31-35) species present, consistent with glucose and mannose trimming via the activities of α-glucosidases and α1,2-mannosidases, respectively, in the endoplasmic reticulum and with the Man_5_GlcNAc_2_ structure being a major checkpoint in *N*-glycan processing (Paschinger et al., 2008; Schachter, 2009; Wilson, 2012). While much of the *N*-glycosylation machinery is conserved, the fucosylation patterns of *C. elegans* are noteworthy as this organism is predicted to express nearly 30 unique fucosyltransferases capable of decorating paucimannose- and oligomannose-type structures not usually observed in higher organisms (Altmann et al., 2001; Cipollo et al., 2002, 2005; Haslam et al., 2002; Haslam and Dell, 2003; Paschinger et al., 2008). Of this type, we detected nine low abundance structures that we have classified as the fucose-rich subgroup (Hex_3−7_dHex_1−4_HexNAc_2_, Figure 2B). Also lower in abundance were truncated complex- and hybrid/complex-type structures that could also be elaborated with one or more fucose residues (Figure 2C). Finally, to assess the comprehensive changes in the *N*-glycome with development, relative glycan abundances were summed for each time point (Figure 2D).

### Charged *O*-Glycan Expression Patterns Peak With Large Worms

**Figure 3:**
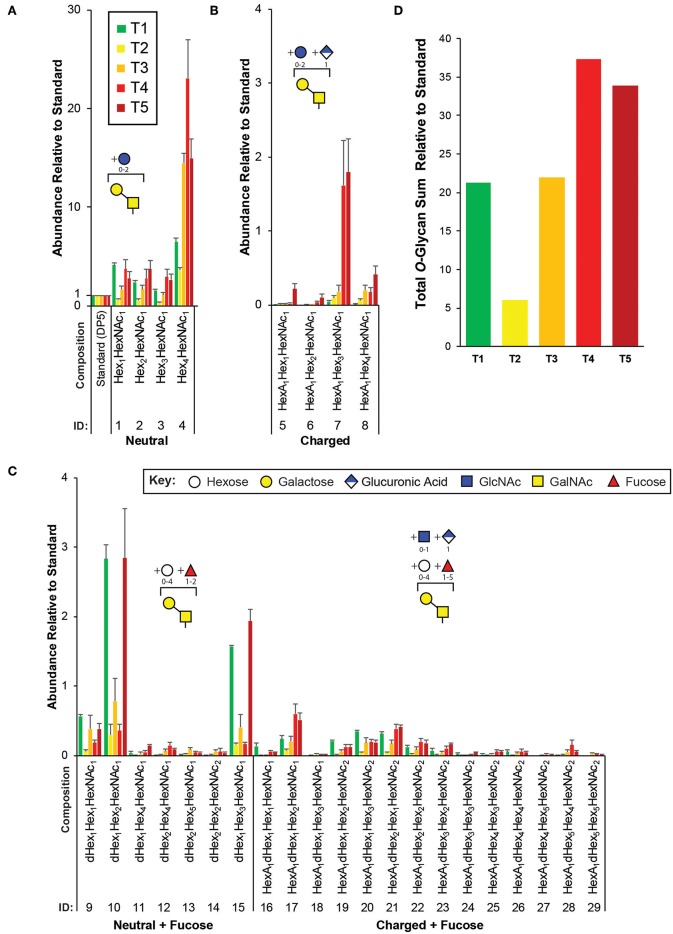
Targeted *O*-glycomics of *C. elegans* at various developmental time points. Presented are the relative abundances of indicated *O*-glycan compositions. Specific *O*-glycan subgroups are indicated in the following panels: **(A)** Neutral **(B)** Charged **(C)** Neutral or Charged + additional Fucoses. Representative cartoon examples of each subgroup structure are shown as insets and displayed in accordance with the SNFG guidelines (Varki et al., 2015). Glycan IDs are presented in Table 1. The sum for the relative *O*-glycan abundances at each time point is presented in **(D)**. Each bar represents the sample mean relative to the internal standard (^13^C-permthylated isomaltopentaose, DP5) where *n* = 7 and the error bars represent SEM.

We next examined changes in the *O*-glycome with development by using a similar targeted approach by generating a list of expected *O*-glycans reported previously for the analysis of LC-MS/MS data (Guerardel et al., 2001; Parsons et al., 2014). Previous *O*-glycomic data for *C. elegans* were generated from mixed stage nematodes. Thus, we report the first analysis of *O*-glycans with respect to discrete sizes of worms. Glycan compositions identified in this study that are most consistent with *O*-glycan subgroups are summarized in Table 1 and Figure 3.

In agreement with previous studies, of the greatest abundance were the neutral, mucin-type core 1 *O*-glycans (Hex_1−4_HexNAc_1_, Figure 3A), followed by neutral *O*-glycans substituted with one or more fucoses (Figure 3C). Relatively minor amounts of charged *O*-glycan species defined by containing HexA (presumably GlcA, Figures 3B,C) with and without additional fucoses or extended are observed. Interestingly, most non-fucosylated charged *O*-glycans, which are lowest in abundance at T1 unlike most of the other glycans identified, appeared to peak in abundance at T4 and/or T5 that contain adult nematodes (Figure 3B). Finally, an evaluation of the total *O*-glycome is presented in Figure 3D.

### Glycans Have Specific Developmental Patterns

**Figure 4:**
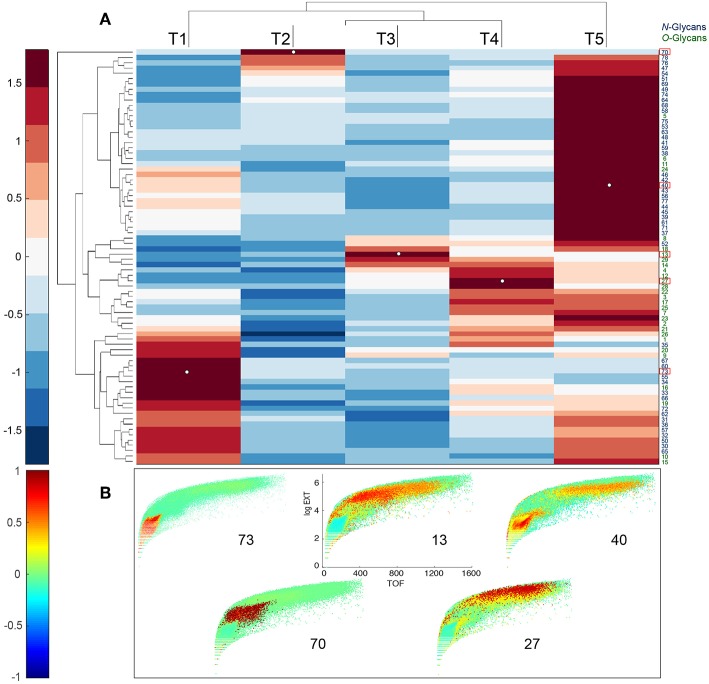
Developmental patterns of glycans in *C.elegans*. **(A)** Upper panel shows heatmap of glycan abundances, together with dendrogram of glycans (rows) and sample timepoints (columns). Glycan abundances were averaged over replicates in the same time point. A color bar of the heatmap is shown on the left. *N*-glycans IDs are shown in black and *O*-glycans are shown in green. **(B)** Lower panels show projections of Pearson correlation coefficients between normalized worm counts and glycan abundances on Biosorter maps. Number on each panel corresponds to the unique glycan, bolded and outlined in red in **(A)**, that is used to calculate the correlation. A color bar of the correlation coefficients is shown on the left.

The heatmap shown in Figure 4A represents an average over each sample within each timepoint (columns) for each glycan (rows). The colors in the heatmap indicate the degree of association between specific glycans and time points. Dark red indicates high levels at that time. We also provide supplementary data (Figure S4) similar to Figure 4A that corresponds to individual replicates before averaging. The tree from HCA on the top of 4A shows that T5 is most closely related to T1, because the T5 samples contained offspring. The other time points group as expected. The tree on the left of 4A groups glycans and indicates structures that are most closely related through biosynthetic steps.

Scheme 1 provides detailed steps used to create Biosorter correlation maps with specific glycans measured from the same samples (Figure 4). The distributions shown in Figure 4B were obtained by using the glycans indicated by numbers as driver peaks. The numbers for each glycan are provided in Table 1 and are also shown on the right-hand side of the glycan heatmap in Figure 4A. We also have shown the driver peaks as small white dots on 4A.

The color scale for the Biosorter correlation maps ranges from 1 to −1, which are Pearson correlation coefficients between the glycan driver and Biosorter position. The regions that are dark red in Figure 4B correlate most highly with that specific driver peak. For example, glycan 73 is only correlated with time point T1, and Table 1 indicates that glycan 73 is HexNAc_1_Hex_5_dHex_2_HexNAc_2_. The color-coding of the numbers to the right of the heatmap in **4A** indicate that glycan 73 is *N*-linked. We tested all of the glycans as driver peaks and found similar distribution patterns within each of the dark red clusters shown in Figure 4A, but we are showing only the specific glycans for clarity.

Clearly, most of the glycans in our study show changes between time points, and we can use the Biosorter distributions to identify glycans that appear in different sizes of worms. For example, glycan 40 (Hex_3_dHex_1_HexNAc_2_) is *N*-linked and is most strongly associated with T1 (L1 stage). However, there is also some correlation with larger animals, and this may indicate that this glycan is expressed embryonically in the gravid adults. Glycan 70 (HexNAc_4_Hex_4_dHex_2_HexNAc_2_) is *N*-linked and is specifically expressed in T2. This glycan has a very low abundance but is clearly associated with a narrow stage of development.

### Correlation Network Between Size, Glycans, and NMR Features

The supernatants of each sample from glycan analyses were also analyzed for metabolites by NMR spectroscopy. As noted above, the 7 replicates from T1 (L1 stage) were combined for NMR, but the other replicates have a one-to-one correspondence with LC-MS and Biosorting data. In this study, we focus exclusively on NMR resonances that statistically correlate with glycan data, as described in methods and shown in Figure S2.

Similar to the approach described in Scheme 1, we first fused the normalized NMR and glycan data in MATLAB and performed SHY analysis (Crockford et al., 2006) by systematically using all of the glycans as driver peaks for correlations in the NMR data (Figure S2). The NMR resonances that correlated with glycans were then binned for network analysis. We attempted to use all the NMR data for this step but were unable to unravel the extensive correlation network that NMR data introduced (data not shown). We used a combination of 2D NMR with COLMARm for database matching and spiking with authentic samples for compound annotation and identification. Table 2 lists NMR metabolites and confidence scores reported in this study.

**Table 2:** NMR.

**Name**	**Cytoscape label^a^**	**Confidence^b^**	**^**1**^H ppm**	**^13^C ppm (COLMARm)^d^**
			**(COLMARm or 1D)^c^**	
UDP-GlcNAc	5.51	5	5.51, 7.93, 5.96, 5.97, 3.73, 4.36, 4.27, 2.06	
Cystathionine	3.13, 3.11	4	2.16, 2.73, 3.10, 3.85, 3.95	32.93, 29.88, 34.76, 56.48, 56.28
Trehalose	3.43, 3.65, 3.87	5	3.44, 3.64, 3.76, 3.84,3.84, 5.19	72.4, 73.76, 63.28, 63.27, 75.21, 95.99
Lactate	4.11	4	4.1, 1.32	71.23, 22.81
Glycerol	3.57, 3.54, 3.63	4	3.56, 3.63, 3.77	65.26, 65.26, 74.85
2-Aminoadipate	3.73	2	3.73, 2.23, 1.88, 1.82, 1.61, 1.65	N/A
Betaine	3.26, 3.90	4	3.26, 3.9	56.05, 68.84
UK-1	5.84		5.83	
Guanosine	5.91	2	5.9. 7.99	
UK-2	5.25, 5.38, 5.55,3.47, 3.70		5.25, 5.38, 5.54, 3.46, 3.69	N/A
Asparagine	2.89	4	2.88, 2.93, 2.99	37.33, 37.33, 54.01
UK-3	5.79		5.79	
Phosphorylcholine	4.17	4	3.19, 3.59, 4.16	56.67, 68.63, 60.83
UK-4	5.38		5.38	
NAD+	9.34	2	9.32, 9.12, 8.82, 8.4,8.19,8.16, 6.08, 6.02	
Glucose-6-phosphate	4.65, 5.23	2	4.64, 5.23, 4.0	
UK-5	9.57		9.57	

a *Obtained from the centers of the interactively binned NMR data*.

b *Confidence scale: (5) verified by spiking; (4) Matches in COLMARm using both HSQC and HSQC-TOCSY; (3) COLMARm matches using HSQC but not HSQC-TOCSY; (2) matches from 1D NMR to literature and/or database libraries; (1) for putatively characterized compounds or compound classes*.

c *For confidence level 4 and trehalose, ^1^H chemical shift values were from COLMARm matched searches; For UDP-GlcNAc, ^1^H shifts from synthetic, spiked standard; confidence level 2, ^1^H shifts from 1D spectra*.

d *For confidence level 4 and trehalose, 13C chemical shift values were from COLMARm matched searches*.

**Figure 5:**
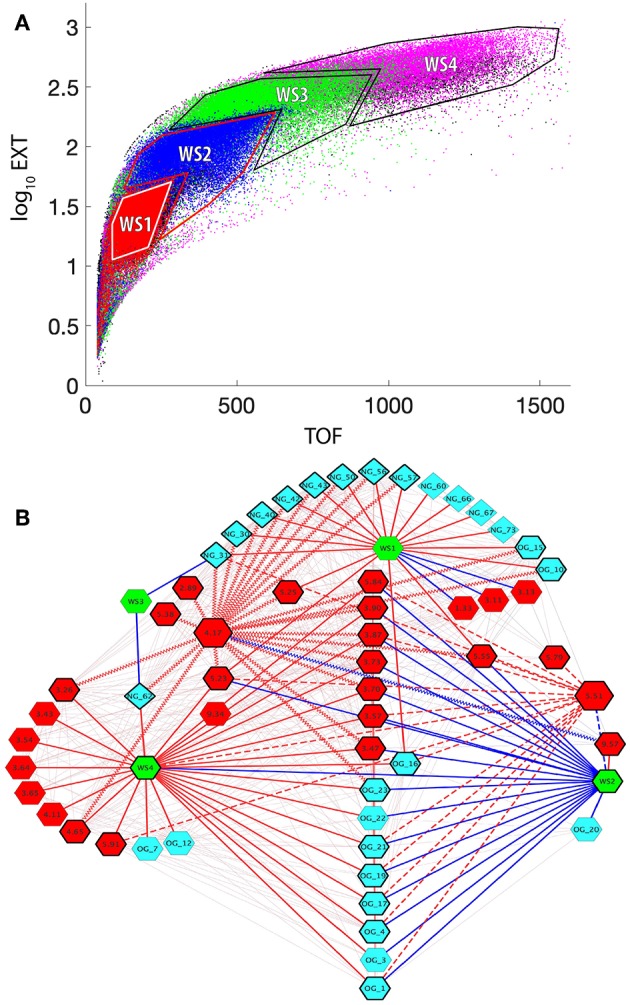
Correlation network between worm sizes, LC-MS-measured glycans, and NMR-measured metabolites. **(A)** Superposition of Biosorter data (Figure S1) for all samples and time points (T1 = red, T2 = blue, T3 = green, T4 = magenta, T5 = black). The data were plotted from T5 on the bottom to T1 on the top to emphasize unique regions. The four distinct resulting regions were binned and labeled for the worm size (WS1-WS4). WS1 corresponds to L1 arrested animals, while the other three stages are less precise. WS4 corresponds to the largest animals in the study and thus are primarily adult. **(B)** Cytoscape correlation network between worm size (WS) regions from **(A)** (green hexagons), N-glycans (teal diamonds), O-glycans (teal hexagons), and NMR data (red hexagons). The numbers for the glycans correspond to Table 1. The numbers for NMR features correspond to chemical shift values from Table 2. Red and blue edges are positive and negative correlation values with a lower threshold of 0.5. The faint lines in the background include all correlations in the network. Solid bold edges connect direct neighbors from WS nodes to glycans and NMR nodes. Wavy bold lines are edges between phosphorylcholine (PC) (4.17 ppm, larger hexagon) and direct neighbors. Dashed bold lines are edges between UDP-GlcNAc (5.51 ppm, larger hexagon) and direct neighbors. The nodes in direct contact with either PC or UDP-GlcNAc are outlined with a bold black line.

Figure 5A shows the regions from the Biosorter distributions that were binned to represent different worm sizes (WS). This binning of sizes allows us to directly compare sizes rather than time points, which contain different mixtures of sizes. As described above, these binned regions cannot be ascribed to specific developmental stages without a more detailed image analysis of animals from each region of the Biosorter distributions, which is beyond the scope of this study. The smallest bin was chosen to overlap with L1 distributions shown in Figure S1, and larger bins correspond to unique Biosorter regions from each time point.

These 3 sets of data—binned Biosorter sizes, glycans, and NMR features that correlate with glycans—were analyzed in Cytoscape to give the correlation network shown in Figure 5B. We adjusted the correlation value and found that *r* = 0.5 allowed for an interpretable number of nodes at each time point. We organized the network around Biosorter size nodes and colored positive and negative correlations as red and blue edges, respectively.

Figure 5B shows the correlation network of worm sizes (green hexagons), N-glycans (teal diamonds), O-glycans (teal hexagons), and NMR features (red hexagons) that correlate with glycans. The glycans have labels “NG_X” or “OG_Y” for N-glycan X or O-glycan Y, with numbers of the glycans from Table 1 or Figure 4A. The NMR resonances are labeled with chemical shift values; assignments (when known) and confidence scores provided in Table 2.

Several aspects of Figure 5B are noteworthy. First, with the exception of NG_62 (N-glycan 62 in Table 1), all N-glycans positively correlate with the smallest worm size (WS1), which corresponds to L1 animals. Only three O-glycans (#10, 15, 16) positively correlate with WS1. Three NMR features negatively correlate with WS1, including cystathionine (3.13 and 3.11 ppm) and lactate (1.33 ppm). Three NMR features positively correlate with WS1, glucose-6-phosphate (5.23 ppm) (overlapped and lower confidence score; Table 2) and two unknowns at 5.25 and 5.55 ppm. The second body size, WS2, has a striking pattern, because with the exception of UK-5 (9.57 ppm), all glycans and NMR features negatively correlate with this size. This includes a large number of O-glycans (#1, 3, 4, 17, 19, 20, 21, 22, 23), all of which except OG_20 also positively correlate to WS4. A group of NMR features negatively correlate with WS2, including UDP-GlcNAc (5.51 ppm), glucose-6-phosphate (5.23 ppm), betaine (3.90 ppm), trehalose (3.87 ppm), 2-aminoadipate (3.73 ppm), glycerol (3.57 ppm), and five unknowns (3.47, 3.70, 5.84, and 5.55 ppm). WS3 is very sparse in the network and only negatively correlates with two N-glycans (#31 and 62). WS4 is the largest body size and largely corresponds with adult animals. WS4 is positively correlated with many of the same O-glycans and NMR features that were negatively correlated to WS2. In addition, WS4 positively correlates to N-glycan 62, two specific O-glycans (#7 and 12), along with several NMR features, including betaine (3.26 ppm), trehalose (3.43, 3.65 ppm), glycerol (3.54, 3.64 ppm), lactate (4.11 ppm), glucose-6-phosphate (5.23 ppm), and guanosine (5.91 ppm).

We highlighted interactions involving two specific NMR features in Figure 5B, UDP-GlcNAc (5.51 ppm) and phosphorylcholine (PC: 4.17 ppm) by bolding the outlines of neighboring nodes and applying thick dashed or zig-zag lines for edges, respectively. UDP-GlcNAc (verified by spiking, Figure S5) positively correlates with several O-glycans (#21, 19, 17, 16, 4, and 1) and a single N-glycan (#31). UDP-GlcNAc negatively correlates with WS2 but positively correlates with WS4 and positively correlates with several other unknown NMR features (5.79, 5.55, 5.84 ppm) as well as glycerol (3.57 ppm), guanosine (5.91 ppm), trehalose (3.87 ppm) and glycose-6-phosphate (5.23 ppm).

PC (4.17 ppm) also has interesting correlation patterns. With the exception of a negative correlation to UK-5 (9.57 ppm), all other correlations are positive. Most notable are several positive correlations to N-glycans (#30, 31, 40, 42, 43, 50, 56, and 57) that are associated with WS1 (L1 animals). It also positively correlates with N-glycan 62, which negatively correlates with WS3 and positively correlates with WS4. PC also positively correlates with the two O-glycans (#10 and 15) that positively associate with WS1. PC positively correlates with several unknown NMR features (5.25, 5.38, 3.70, 3.47, and 5.55 ppm), asparagine (2.89 ppm), glucose-6-phosphate (4.65 and 5.23 ppm), betaine (3.26 and 3.90 ppm), trehalose (3.87 ppm), and 2-aminoadipate (3.73 ppm).

## Discussion

Our study has combined three different types of data collected from the same samples of *C. elegans*: Biosorter data, LC-MS-detected glycans, and NMR-detected metabolites. Important technical steps were implementing a protocol that samples a small percentage of a culture for Biosorting and utilizing the pellet after homogenization for glycan analysis. This provides the ability to conduct statistical correlations of different data types using the same biological replicates. Although interesting data were obtained individually, the most important findings were when data were combined.

The protocol to correlate Biosorter data with analytical data provided a key link in our study. Importantly, it is not limited to the LC-MS/MS glycan data. Any quantitative analytical data can be substituted for the glycan data outlined in Scheme 1. We think that the approach correlating Biosorter-based population distribution data will allow much more detailed omics studies in worms or other organisms like zebrafish or drosophila embryos, which can also be Biosorted. Although we have not yet examined the detailed biological replicate requirements, we are confident that developmental stage information can be extracted from mixed cultures by combining image analysis of worms isolated from different Biosorter regions, binning those Biosorter regions, and using them as driver peaks for a variety of quantitative omics data, including RNAseq, proteomics, and untargeted LC-MS metabolomics. Moreover, we see no reason why this approach could not also be used in flow cytometry analysis of cells, which would make it even more broadly applicable.

By utilizing previously identified glycan compositions reported in *C. elegans*, we have carefully analyzed released and permethylated *N*- (Figure 2) and *O*-glycans (Figure 3) using a high-throughput strategy by LC-MS/MS. The relative abundances for certain *N*-glycans compositions were consistent with previous reports, as described above (Cipollo et al., 2002; Haslam et al., 2002; Natsuka et al., 2002; Paschinger et al., 2008; Geyer et al., 2012). In particular, to assess global changes in the total *N*-glycome with development, relative glycan abundances were summed for each developmental time point (Figure 2D). In agreement with Cipollo et al. (2005), *N*-glycan abundances follow the trend of being highest in the early L1 larval stages (T1 and mix with T5) with relatively lower amounts within the intermediate time points (T2, T3, and T4). Thus, our results recapitulate the dynamic *N*-glycan utilization during development with approximately 4- to 8-fold differences in total *N*-glycan abundances when comparing L1 to intermediate stages. This trend is even more striking when we use unique regions of the Biosorter distribution to correlate worm size with glycans (Figure 5B), because nearly every *N*-glycan is positively correlated with the smallest L1 animals.

An NMR resonance from phosphorylcholine (PC) (4.17 ppm, Figure 5B) was correlated with the N-glycans (#30, 31, 40, 42, 43, 50, 56, and 57) that were correlated to L1 animals (WS1, Figure 5B) and the single *N*-glycan (#62) that was correlated with the largest size worms (WS4, Figure 5B). It has been previously demonstrated that *N*-Glycans of *C. elegans* can be elaborated by PC, and that this modification is stage specific, being detected in L1, L4, adult, and dauer (Cipollo et al., 2002, 2004, 2005), in good agreement with our study. While we did not search for any low abundance PC-substituted *N*-glycans, PC does cluster with the *N*-glycan subgroups that may be modified with it, which suggests that the highly correlated *N*-glycans we identified by LC-MS/MS may be potential acceptor substrates in which PC modifies by a yet-to-be-identified transferase. Furthermore, the developmental PC correlation may be significant to the modification of glycolipids, which were not analyzed in this report, but have been implicated in specific stages of development and embryogenesis (Gerdt et al., 1999).

The total *O*-glycan relative abundances followed a different pattern than *N*-glycans during development, with the approximate pattern T4 ≈ T5 > T1 ≈ T3 > T2 (Figure 3D). As this pattern would be mostly dominated by the most abundant structures, superficial inspection shows the general trend holds true with most glycans identified in this study, except for several low abundance fucosylated neutral glycans (#9, 10, 13, and 15) that followed a pattern with the greatest levels in T1, T3, and T5 (Figure 3C). The pattern of *O*-glycan correlation with worm sizes in Figure 5B shows a complex pattern in which several *O*-glycans are negatively correlated with WS2 but positively correlated with WS4. This pattern suggests a switch during development from smaller to larger worms. In most animals, a null deletion of OGT—the glycosyltransferase responsible for adding *O*-GlcNAc to proteins (Haltiwanger et al., 1990, 1992)—is embryonic lethal. However, in *C. elegans, ogt-1* null animals are viable, and these animals accumulate UDP-GlcNAc (Rahman et al., 2010; Ghosh et al., 2014), which is the substrate for OGT. Moreover, the modENCODE (Celniker et al., 2009) expression of *ogt-1* RNA reported on WormBase (Stein et al., 2001) shows a steady decline of expression from the highest levels in early embryonic stages to the lowest in L4 and young adult. These observations are both consistent with the network in Figure 5B, which shows UDP-GlcNAc levels measured by NMR positively correlate with many of the same *O*-glycans that have the reciprocal correlation pattern to WS2 and WS4. This suggests a relationship between increases in UDP-GlcNAc and decreases in *ogt-1* gene expression. Perhaps this represents a switch from primary utilization of *O*-GlcNAc for dynamic protein *O*-GlcNAc-ylation mediated by OGT at earlier stages to the biosynthesis of complex mucin-like *O*-glycans at adult stages. In more complex *O*-glycan biosynthesis, UDP-GlcNAc is converted to UDP-GalNAc by UDP-*N*-acetylglucosamine 4'-epimerase (GalE). We examined our NMR data for UDP-GalNAc but were unable to unambiguously assign it. A likely NMR resonance of guanosine (5.91 ppm, Figure 5B; Table 2) positively correlates with large worms (WS4) and UDP-GlcNAc, suggesting a relationship between the *O*-glycans that positively correlate with the same large worms. GDP-fucose is the sugar nucleotide donor for fucosyltransferases that generate these structures. GDP-fucose is synthesized from GDP-mannose in *C. elegans* (Rhomberg et al., 2006). Fucosylation is required for normal development (Menzel et al., 2004).

Future studies will aim to adapt our workflow to analyze natively methylated structures by using ^13^C-permethylation. In this report, we have broadly established the dynamic *N*- and *O*-glycome, and these data could be utilized in tracking dynamic changes in the glycoproteome or of key glycoproteins throughout developmental transitions including O-GlcNAc modified proteins. While the work presented here represents a pilot study for combining different omic data to better understand development in *C. elegans*, it could easily be expanded/adapted to capture additional –omic datasets (transcriptomic, proteomic, and lipidomic) and/or applied to studies aimed at uncovering the impact of genetic and environmental perturbations.

## Author Contributions

MS conducted the glycan measurements. FT conducted NMR measurements and analysis. SZ developed algorithms for Biosorting correlation analysis and conducted Cytoscape analysis. MJ developed interactive NMR binning and assisted with Cytoscape analysis. FP made worm samples. DW analyzed the glycomics data. LW, AE, MS, FT, and SZ advised on design and interpretation. MS, LW, and AE wrote the initial manuscript draft, which was edited by all authors and finalized by LW and AE. All authors approved the final version.

### Conflict of Interest Statement

The authors declare that the research was conducted in the absence of any commercial or financial relationships that could be construed as a potential conflict of interest.

## Abbreviations

*C. elegans*: Caenorhabditis elegans
dHex, deoxyhexose, namely Fuc: fucose
Gal: galactose
GalNAc: *N*-acetylgalactosamine
GDP-Fucose: Guanosine diphosphate fucose
Glc: glucose
GlcA: glucuronic acid
GlcNAc: *N*-acetylglucosamine
HBP: Hexosamine Biosynthetic Pathway
HCA: Hierarchical Clustering Analysis
Hex, hexose, either Glc, Gal: or Man
HexA, hexuronic acid: namely GlcA
HexNAc, *N*-acetylhexosamine: either GlcNAc or GalNAc
LC-MS/MS: liquid chromatography-mass spectrometry and tandem mass spectrometry
Man: mannose
NMR: Nuclear Magnetic Resonance
*O*-GlcNAc: *O*-linked β-*N*-acetylglucosamine
OGT: *O-*GlcNAc Transferase
PC: phosphorylcholine or *O*-phosphocholine
PNGase A: Protein *N*-Glycosidase A
STOCSY: Statistical total correlation spectroscopy
UDP-GlcNAc: Uridine diphosphate *N*-acetylglucosamine.
