# Supplementary material for: Correlations Between LC-MS/MS-Detected Glycomics and NMR-Detected Metabolomics in Caenorhabditis elegans Development
Source: Front Mol Biosci. 2019 Jun 28;6:49. doi: 10.3389/fmolb.2019.00049 (PMC6611444; doi:10.3389/fmolb.2019.00049)
Supplement: Supplementary file 1 [file Data_Sheet_1.PDF]

# Supplementary Material

# Correlations between LC-MS/MS-detected Glycomics and NMR-detected Metabolomics in *Caenorhabditis elegans* Development.

**M. Osman Sheikh[1,†], Fariba Tayyari[1,2,3,†], Sicong Zhang[1,2†], Michael T. Judge[1,4], D. Brent Weatherly[1], Francesca V. Ponce[1], Lance Wells[1,2,*] , and Arthur S. Edison[1,2,3,4*]**

[1] Complex Carbohydrate Research Center, University of Georgia, Athens, Georgia, USA.
[2] University of Georgia, Department of Biochemistry & Molecular Biology, Athens, Georgia, USA.
[3] University of Georgia, Institute of Bioinformatics,
[4] University of Georgia, Department of Genetics, Athens, Georgia, USA.

*** Correspondence:**
Dr. Lance Wells
lwells@ccrc.uga.edu

Dr. Arthur S. Edison
aedison@uga.edu

[†]These authors contributed equally

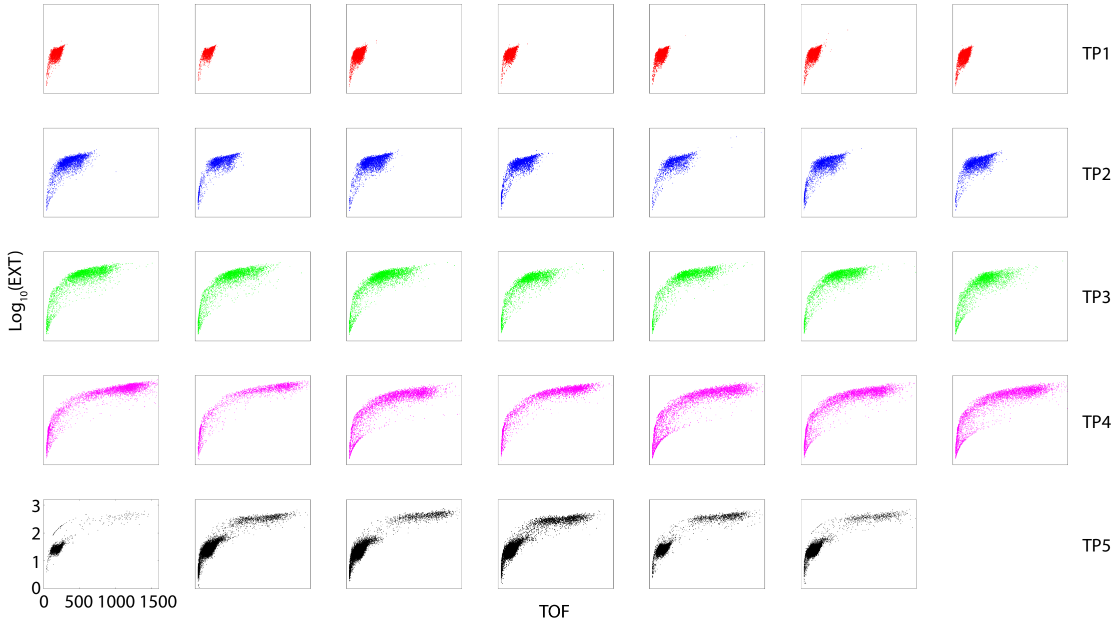


**Figure S1. Individual *C.elegans* distribution plots for each sample.** Each subplot shows raw reads from the Union Biometrica Biosorter for each sample. The biological replicates are the columns. Different colors indicate samples collected from different time points (red: TP1, blue: TP2, green: TP3, magenta: TP4, black: TP5). One replicate at TP5 was not biosorted.

Figure S2A

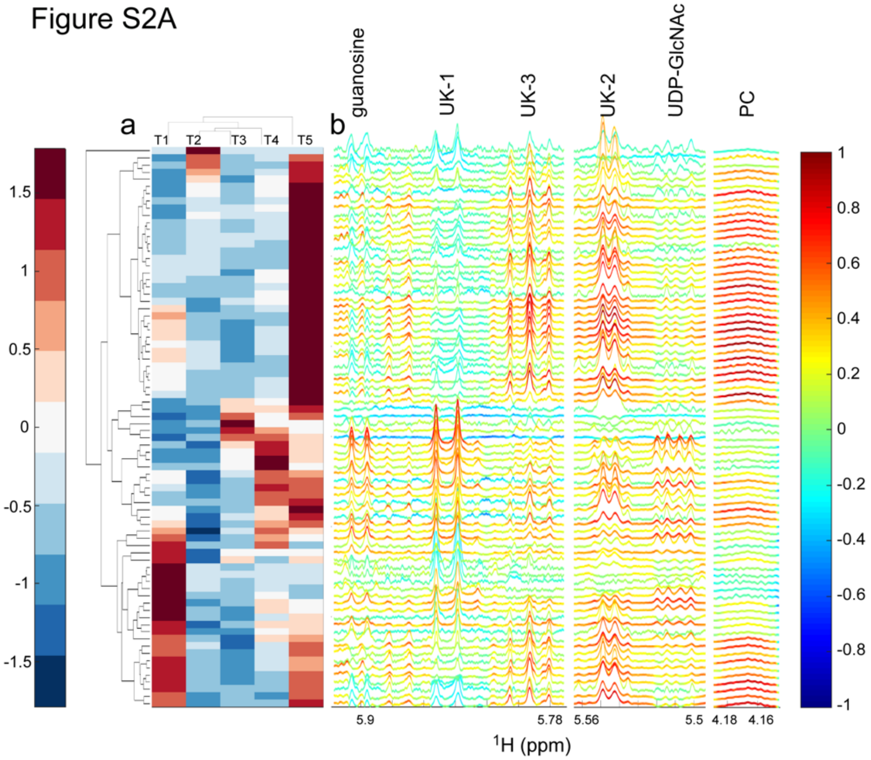


**Figure S2A. Correlations between NMR-measured metabolites and LC-MS-measured glycans.** (**a**) Heatmap of glycan abundances and dendrogram of glycans (rows) and sample time points (columns) (same heatmap as used in Figure 4). Glycan abundances were averaged over replicates. A color bar of the heatmap is shown on the left. (**b**) Regions of NMR STOCSYs on glycans. A color bar of the correlation coefficients is shown on the right.

Figure S2B

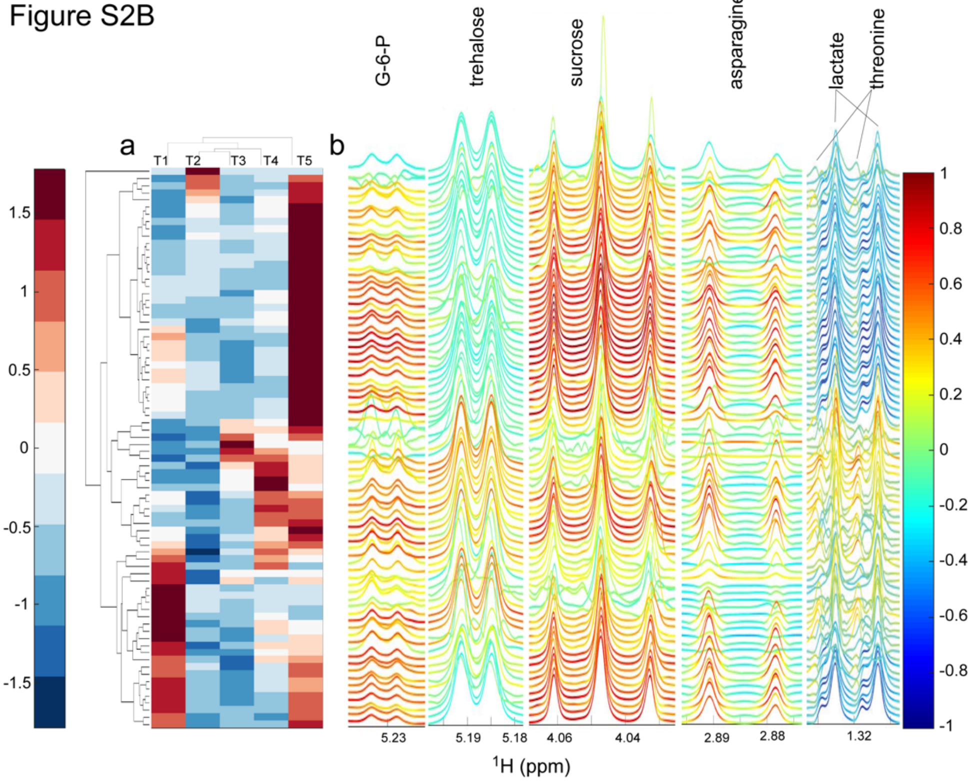


**Figure S2B.** See caption for S2A.

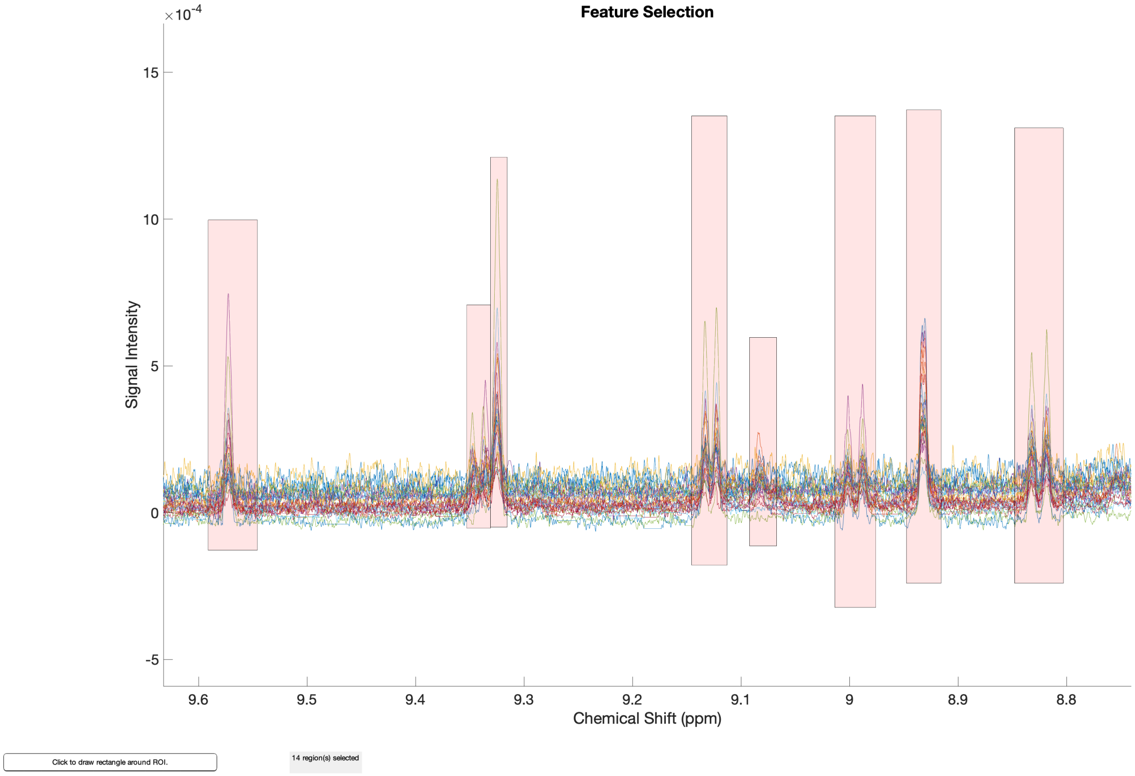


**Figure S3: NMR interactive binning example.** The interactive binning algorithm described in 2.6 allows for user-defined regions with variable width and range to be specified for binning. This method was used to extract features for Figure 5B. The boxes below the figure are part of the interactive GUI. This expansion shows 8 of the 14 total regions selected from this particular session. The complete workflow is available through the Edison lab GitHub site (https://github.com/artedison/Edison_Lab_Shared_Metabolomics_UGA).

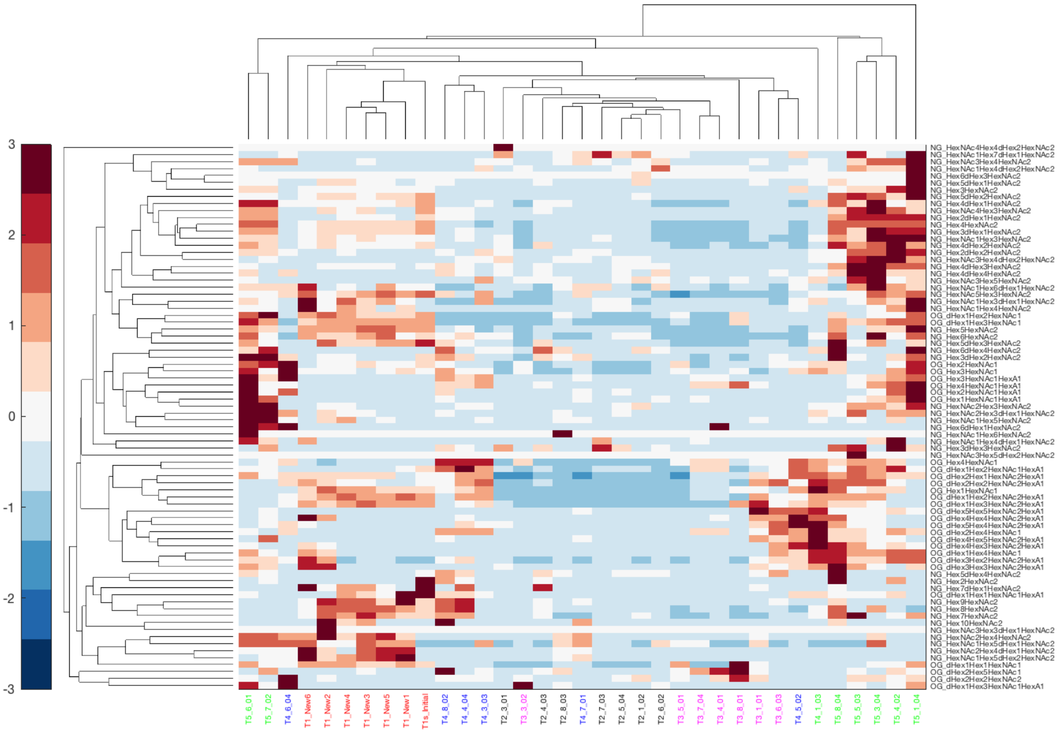


**Figure S4. Developmental patterns of glycans in *C. elegans*.** The heatmap shows glycan abundances, together with dendrogram of glycans (rows) and sample timepoints (columns). This is similar to Figure 4 in the main text, but each column is a biological replicate rather than the time average. A color bar of the heatmap is shown on the left.

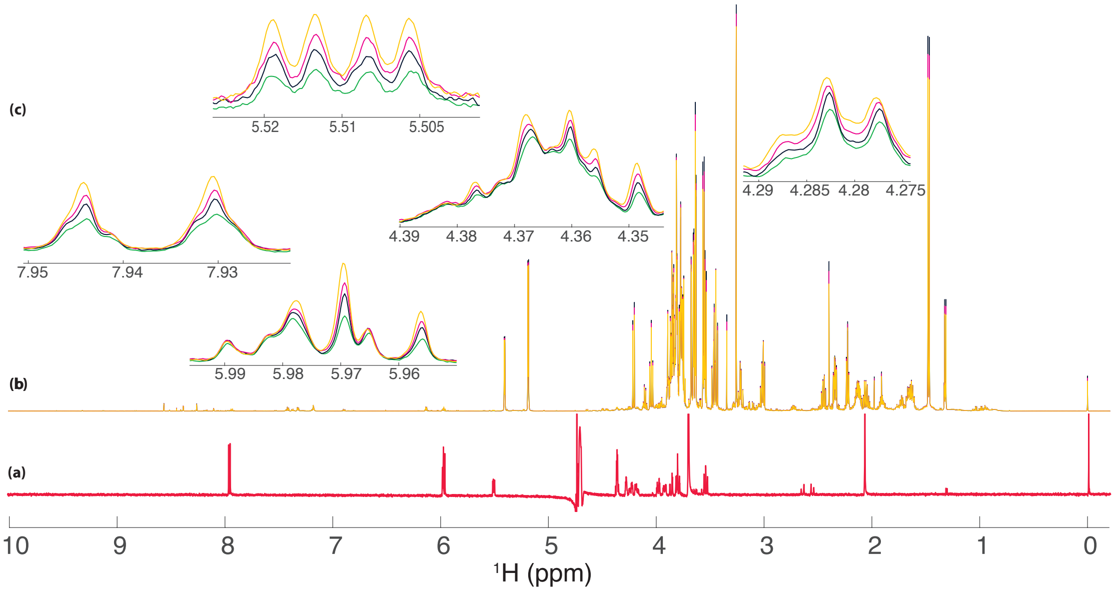


**Figure S5. Spiking of UDP-GlcNAc.** Spectrum of synthetic UDP-GlcNAc (a; red); overlay of all 29 experimental worm samples at different time points (b), and different regions after one of the T4 samples was spiked with the UDP-GlcNAc standard solution (green: no spiking; dark blue: first spike; magenta: second spike; yellow: third spike.)
